# Evaluation of visible implant elastomer tags in zebrafish (*Danio rerio*)

**DOI:** 10.1242/bio.20136460

**Published:** 2013-11-11

**Authors:** Claudia Hohn, Lora Petrie-Hanson

**Affiliations:** Department of Basic Sciences, College of Veterinary Medicine, Mississippi State University, PO Box 6100, Starkville, MS 39762-6100, USA

**Keywords:** Identification, Zebrafish, *Danio rerio*, Visible implant elastomer, Tagging, VIE tag

## Abstract

The use of the visible implant elastomer (VIE) tagging system in zebrafish (*Danio rerio*) was examined. Two tag orientations (horizontal and vertical) at the dorsal fin base were tested for tag retention, tag fragmentation and whether VIE tags affected growth and survival of juvenile zebrafish (1–4 month post hatch). Six tag locations (abdomen, anal fin base, caudal peduncle, dorsal fin base, pectoral fin base, isthmus) and 5 tag colors (yellow, red, pink, orange, blue) were evaluated for ease of VIE tag application and tag visibility in adult zebrafish. Long-term retention (1 year) and multiple tagging sites (right and left of dorsal fin and pectoral fin base) were examined in adult zebrafish. Lastly, survival of *recombination activation gene 1^−/−^* (*rag1^−/−^*) zebrafish was evaluated after VIE tagging.

The best tag location was the dorsal fin base, and the most visible tag color was pink. Growth rate of juvenile zebrafish was not affected by VIE tagging. Horizontal tagging is recommended in early stages of fish growth (1–2 months post hatch). VIE tags were retained for 1 year and tagging did not interfere with long-term growth and survival. There was no mortality associated with VIE tagging in *rag1^−/−^* zebrafish.

The VIE tagging system is highly suitable for small-sized zebrafish. When familiar with the procedure, 120 adult zebrafish can be tagged in one hour. It does not increase mortality in adult zebrafish or interfere with growth in juvenile or adult zebrafish.

## Introduction

Zebrafish, *Danio rerio*, is a popular animal model and the number of mutants available is rapidly increasing (Kettleborough et al., 2013). Many mutant strains are of the same phenotype and have to be kept in separate tanks requiring large rearing facilities and substantial financial support (Sire et al., 2000). In addition, many zebrafish facilities have multiple users increasing the possibility of accidentally mixing different broodstock which could go unnoticed and render years of research useless. In our facility we have been using visible implant elastomer (VIE) tags to batch mark broodstock of different zebrafish strains. The elastomer is an inert, non-immunogenic polymer that is injected under the skin. Multiple colors are available, and they are easily discerned by the unaided eye. Visible implant elastomer (VIE) tags have been successfully used for many aquatic species (Northwest Marine Technology, Inc., 2008, ‘Visible implant elastomer tag project manual’, http://www.nmt.us/support/appnotes/ape06.pdf) (Blackburn et al., 2011; Cousin et al., 2012) but the use in zebrafish to date is sparse. VIE tags need to be validated for each species under study (Astorga et al., 2005) and in this study we evaluated batch tagging of wild-type and *rag1^−/−^* zebrafish. Tag position, tag color and tag loss rate are important parameters to investigate before the routine use of VIE tags can be recommended for a species (Close and Jones, 2002; Astorga et al., 2005). In this study we investigated these basic parameters and compared tagging sites and survival after elastomer injection in zebrafish.

## Materials and Methods

### Animal care

Wild-type and *rag1^−/−^* mutant zebrafish (Tübingen strain) were housed in the Mississippi State University College of Veterinary Medicine (MSU-CVM) specific pathogen free (SPF) fish hatchery (Hohn and Petrie-Hanson, 2007; Petrie-Hanson et al., 2009). Fish were propagated according to modified standard protocols posted at http://www.cvm.msstate.edu/zebrafish/index.html. The Institutional Animal Care and Use Committee at Mississippi State University approved all experimental animal protocols.

In all trials, zebrafish were anesthetized in 110 mg/L buffered tricaine methane sulfonate (MS222). Visible implant elastomer tags were injected using an insulin syringe. After recovery from anesthesia, fish were moved to 20 L tanks in a flow-through water system (approximately 0.5 L min^−1^) and maintained at 27±1°C. Tanks were aerated to maintain oxygen levels between 6.0 and 6.5 mg L^−1^. Since needle wounds take several days to heal, fish were not handled for the recommended time of at least 10 days post VIE injection (Northwest Marine Technology, Inc., 2006, ‘Tagging small fish with visible implant elastomer’, http://www.nmt.us/support/appnotes/ape03.pdf). All fish were held under the same conditions during all experiments and were observed 3 times a day for tag loss and health status. Moribund fish were euthanatized in 340 mg/L MS222, and mortalities were recorded for the duration of the experiment.

### VIE tags

A manual injection kit (60 mL) for VIE tags was purchased from Northwest Marine Technology, Inc. (Shaw Island, WA, USA, http://www.nmt.us). Elastomer was prepared according to kit instructions for minimal volume (mixing small quantities of VIE) (Northwest Marine Technology, Inc., 2011, ‘Manual elastomer injection systems. Instructions for 10:1 visible implant elastomer’, http://www.nmt.us/products/vie/manual_vie_instructions.pdf). A small amount of elastomer of each mixed batch was expressed onto aluminum foil to determine proper curing. To prevent loss of VIE tag the syringe needle was inserted 2 mm deeper into the tissue then the intended length of the elastomer tag, elastomer was expressed and the needle retracted until about 1 mm before the injection site and the needle was withdrawn from the fish leaving at least 1 mm after the injection site without elastomer. In trial 4 and 5 VIE injection times were recorded. The VIE injection time includes capturing the fish, tagging the fish and releasing it back into the tank.

### Trial 1: VIE tag location

**Fig. 1.**
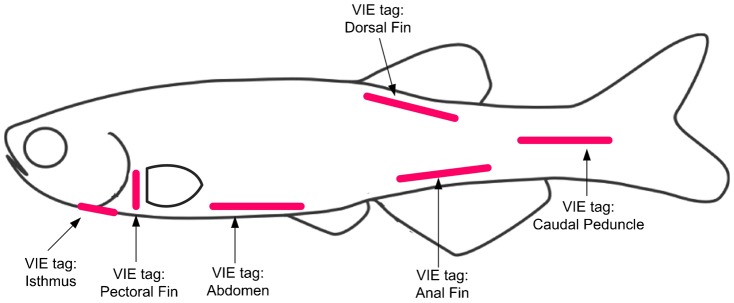
Visible implant elastomer (VIE) tags. VIE tags were evaluated at six positions in zebrafish as indicated by arrows.

To determine optimal tagging sites, groups of 10 adult zebrafish were implanted with a 4 mm strip of pink elastomer at one of the following sites: abdomen, anal fin base, caudal peduncle, or dorsal fin base and with a 2 mm strip of pink elastomer at the pectoral fin base or isthmus (Fig. 1). Fish groups were held for 20 days, after which tag retention was determined at each site. Tag location was evaluated by ease of elastomer application (easy/difficult), retention of tag (tag retained/tag partially retained/tag lost) and wound healing (fully healed/not fully healed).

### Trial 2: VIE tag color and visibility

To determine optimal tag color, 2 injection sites (dorsal fin base and pectoral fin base) were separately evaluated with 10 adult zebrafish per site and color (yellow, red, pink, orange, blue). Fish were implanted with approximately 4 mm of elastomer of one color at the dorsal fin base or with approximately 2 mm of elastomer of one color at the pectoral fin base. Fish groups were held for 20 days, after which tag color visibility was determined at each site.

### Trial 3: Growth study

Fragmentation, poor visibility and loss of elastomer tags are most likely during the major growth phase of fish (Astorga et al., 2005). In zebrafish the main growth phase is between hatching and 3 months post hatch (mph). VIE tags were injected into 1 mph, 2 mph and 3 mph juveniles and zebrafish were evaluated for tag fragmentation (number of fragments), tag retention (tag retained/tag lost), and fish growth, weight and survival. Since fish grow in length and width, horizontal and vertical tags at the dorsal fin base were evaluated. For each treatment, 30 fish were injected with horizontal (5 mm) VIE tags, 30 fish were injected with vertical (3 mm) VIE tags and 30 fish served as control. Fish length and weight were determined at the beginning and end of the experiment. Ten fish per tank were held for one month.

### Trial 4: Long-term retention study

To determine the long-term retention of VIE tags, 50 female and 50 male adult zebrafish were injected at the dorsal fin base with pink elastomer and 50 female and 50 male fish served as controls. Fish length and weight were determined at the beginning and end of the experiment. Injection time and elastomer quantity were recorded. Fish were held for 1 year as broodstock and were spawned twice per month. Survival and tag retention were recorded after one year.

### Trial 5: Multiple tagging sites

To determine survival and growth of adult zebrafish tagged at multiple sites, 10 female and 10 male adult (4 mph) zebrafish were injected at 2 sites (right and left of dorsal fin), 10 female and 10 male adult (4 mph) zebrafish were injected at 3 sites (right and left of dorsal fin and pectoral fin base), and 10 fish served as controls. Fish were held at a density of 10 fish/20 L. Fish length and weight were determined at the beginning and end of the experiment. Injection time was recorded and tag retention was determined after 1 month.

### Trial 6: Survival of rag1^−/−^ zebrafish

To determine survival of immune-suppressed *rag1^−/−^* zebrafish, 30 adult (4 mph) *rag1^−/−^* and 30 wild-type zebrafish were injected with VIE tags at the dorsal fin base. Ten fish/tank were held for 1 month and evaluated.

### Statistical methods

Treatment groups for each trial are described above and are summarized in Table 1. Number of tanks per treatment and number of fish per tank are described above. Mortality data and tag retention of treatments between groups were analyzed by contingency table analysis using a chi-square test of independence for comparison with a level of significance at *P*≤0.05. Growth data were analyzed by one-way analysis of variance (ANOVA) with a post hoc Tukey analysis for multiple comparisons with a level of significance at *P*≤0.05. Tag fragmentation was analyzed by independent sample T-test with a level of significance at *P*≤0.05. Statistical analyses were performed using SPSS for Windows 15.0 (SPSS Inc., Chicago, IL).

**Table 1.**
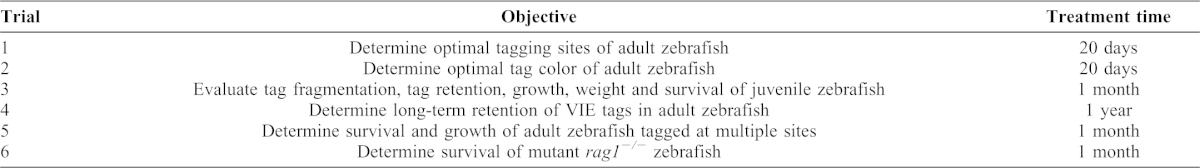
Summary of VIE tagging trials.

## Results

### Trial 1: VIE tag location

In Trial 1, injection site healing, ease of application and elastomer retention were evaluated. Table 2 provides an overview of all VIE tag locations tested. The isthmus was a difficult injection site and only a small amount of elastomer could be injected. All elastomers were retained, but were difficult to see. The pectoral fin base also retained all VIE tags but was difficult to inject and if not carefully done could fatally injure the fish. The dorsal fin base elastomer injections were easy, all tags were retained and the injection site healed well. Therefore the dorsal fin base is the preferred location for VIE tagging in zebrafish. Overall, the best injection sites were the dorsal fin base followed by the pectoral fin base. All tags in the dorsal and pectoral fin bases were retained 4 months after the initial injections.

**Table 2.**
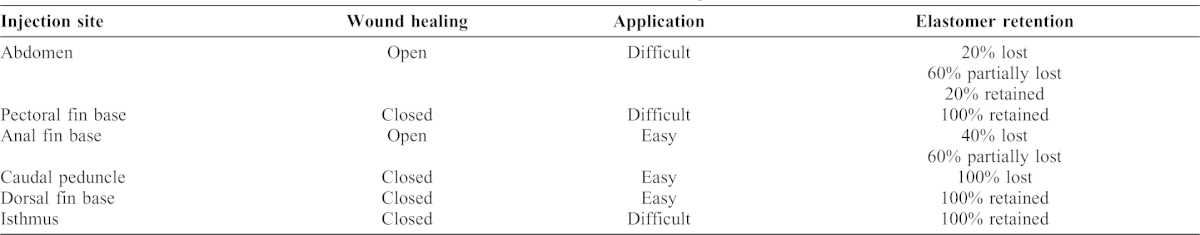
Evaluation of VIE tag location.

### Trial 2: VIE tag color and visibility

When VIE tag colors were compared (at the dorsal fin bases and pectoral fin bases), all tags were retained and injection sites were healed when fish were evaluated after 20 days. Yellow and orange as well as pink and red VIE tags were difficult to distinguish from each other and blue was not visible in ambient light but was visible when fluoresced by UV light. Visibility of tags was better at the pectoral fin base but fish had to be removed from the water to be examined whereas tags at the dorsal fin base were visible without removing the fish from water. A ranking of the most to least visible colors was pink, red, orange, yellow and blue.

### Trial 3: Growth study

**Fig. 2.**
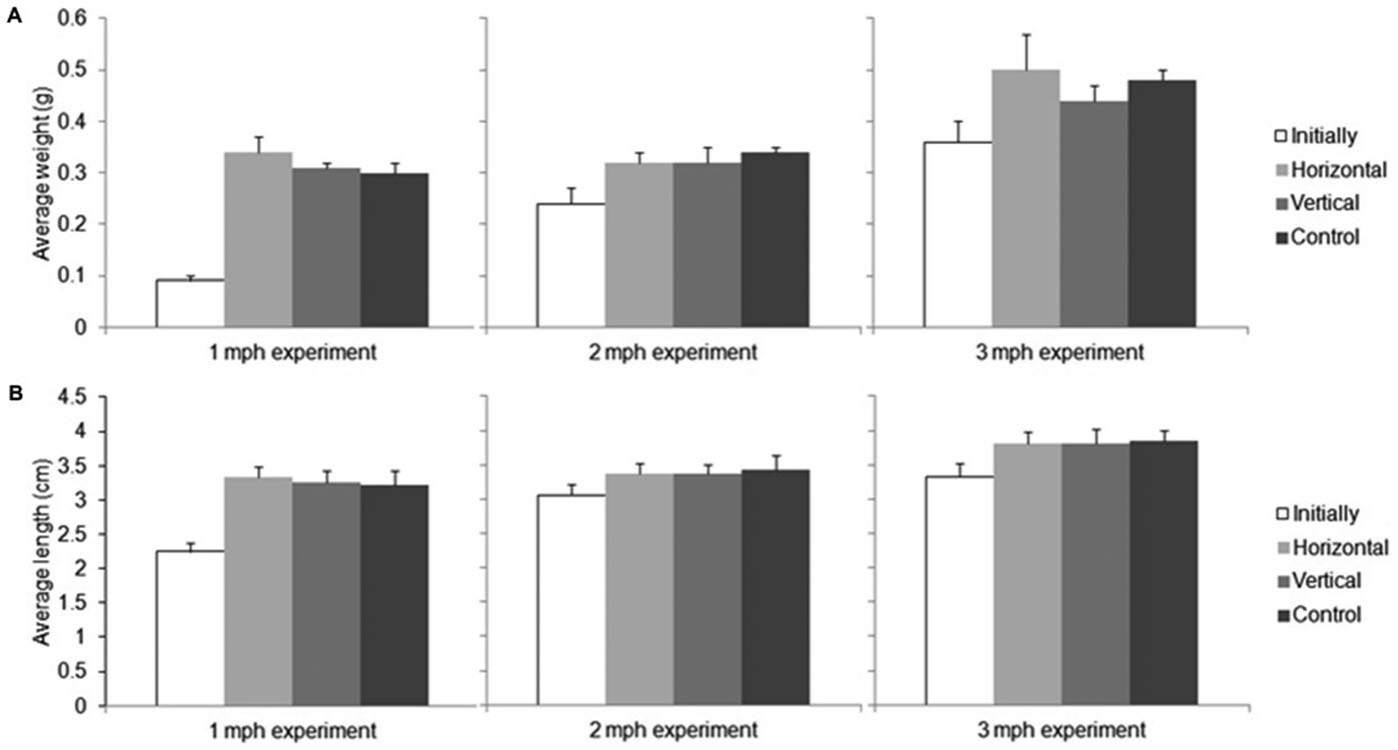
Summary of growth data Trial 3. Juvenile zebrafish were weighed (mean ± sd) and length was measured (mean ± sd) at the start of an experiment (Initially) and at the end of an experiment (Horizontal, Vertical, Control). There was no significant difference in weight at the end of the 1 mph, 2 mph and 3 mph experiments (panel A). There was no significant difference in length at the end of the 1 mph, 2 mph and 3 mph experiment (panel B). The growth rate of juvenile zebrafish was not affected by tagging at the dorsal fin. Chi square test *P*>0.05 (mph  =  months past hatch).

Fish were injected with VIE tags during their main growth phase and the effect of the tag on growth, survival (Fig. 2), tag fragmentation and retention was evaluated. We determined from earlier studies (data not shown) that fish as young as 1 mph can be injected with VIE tags. At 1 mph juvenile zebrafish weighed 0.09±0.01 g and were 2.25±0.12 cm long. After one month survival was 100% and the growth rate between horizontally tagged (0.34±0.03 g and 3.33±0.16 cm), vertically tagged (0.31±0.01 g and 3.26±0.16 cm) and control (0.3±0.02 g and 3.22±0.21 cm) fish was not significantly different (ANOVA weight: *F*(2,6) = 4.19, *P* = 0.073 and length: *F*(2,88) = 2.85, *P* = 0.064) (Fig. 2). Tag retention was 90% in the horizontal tags and 37% in the vertical tags showing that at between 1 and 2 mph tag retention is significantly greater if juvenile zebrafish are injected with horizontal tags (c^2^(1,*N* = 60) = 16.48, *P*<0.001). Fragmentation was not significantly different between the horizontal tags and the vertical tags (2.08 versus 2 fragments respectively, *t*(34) = 0.271, *P* = 0.669). At 2 mph zebrafish weighed 0.24±0.03 g and were 3.06±0.15 cm long. When horizontally injected, 2 fish died 1 day post injection (93% survival rate). For all other treatments, 100% of the fish survived and there was no significant difference in survival and growth rate between horizontally tagged (0.32±0.02 g and 3.37±0.15 cm), vertically tagged (0.32±0.03 g and 3.37±0.13 cm) and control (0.34±0.01 g and 3.43±0.21 cm) fish (ANOVA weight: *F*(2,6) = 2.85, *P* = 0.135 and length: *F*(2,88) = 1.18, *P* = 0.312) (Fig. 2). Tag retention was not significantly greater in the horizontal tags than in the vertical tags (83% versus 84% respectively) (c^2^(1,*N* = 60) = 0.577, *P* = 0.448). Fragmentation was not significantly different between the horizontal tags and the vertical tags (2.6 versus 2.4 fragments respectively, *t*(45) = 1.0, *P* = 0.528). At 3 mph juvenile zebrafish weighed 0.36±0.04 g and were 3.33±0.2 cm long. After one month 100% of fish in all treatments survived and there was no significant difference in growth rate between horizontally tagged (0.5±0.07 g and 3.8±0.19 cm), vertically tagged (0.44±0.03 g and 3.8±0.22 cm) and control (0.48±0.02 g and 3.85±0.15 cm) fish (ANOVA weight: *F*(2,6) = 1.33, *P* = 0.332 and length: *F*(2,88) = 0.151, *P* = 0.86) (Fig. 2). Tag retention was not significantly different between the horizontal tags versus the vertical tags (97% versus 94% respectively) (c^2^(1,*N* = 60) = 1.964, *P* = 0.161). Fragmentation was significantly different between the horizontal tags and the vertical tags (1.5 versus 1 fragment respectively, t(55) = 3.38, *P* = 0.01).

In conclusion, growth rate of juvenile zebrafish was not affected by tagging at the dorsal fin (Fig. 2). Retention of horizontal tags was better than retention of vertical tags in early stages of fish growth (1–2 mph).

### Trial 4: Long-term retention study

When 4 month old female zebrafish were implanted, average fish weight was 0.7±0.03 g. After one year, average weight increased to 1.1±0.12 g; that is comparable to the average weight of 1.0±0.16 g in control fish. Survival rates were 96% in tagged females and 94% in untagged female zebrafish. Male zebrafish weighed 0.45±0.05 g at 4 mph. After one year, average weight increased to 0.63±0.07 g; that is comparable to the average weight of 0.64±0.12 g in control fish. The survival rate of tagged male zebrafish was 90% compared to 94% in untagged male fish. All fish retained their VIE tag for the duration of the study. Tagging time and elastomer use were recorded to minimize wastage of elastomer material. On average it took 22±3 seconds to inject a female zebrafish with a 5 mm strip VIE elastomer and 29±6 seconds to perform the same procedure in male zebrafish. A total of 0.1 mL of VIE elastomer was used to mark 50 fish with one tag. In summary, tagging does not interfere with long-term growth and survival. Fecundity of fish was not affected by VIE tags and on average, 120 adult zebrafish can be injected with one tag in one hour.

### Trial 5: Multiple tagging sites

Adult zebrafish were injected with 2 or 3 VIE tags to assess survival, growth and tag retention. One month after tagging, survival was 100% in all treatments. One month after tagging, survival was 100% in all treatments. Eighty percent of male zebrafish that received 2 caudal VIE tags retained both tags. Also, growth was comparable to control fish in which pre-tagging weight was 0.45±0.02 g per fish, male zebrafish with tags weighed 0.47±0.01 g and control fish weighed 0.47±0.02 g. Seventy percent of female zebrafish that received 2 caudal VIE tags retained both tags. Growth was comparable to control fish in which the average pre-tagging weight was 0.69±0.05 g, female zebrafish with tags weighed 0.68±0.3 g and control fish weighed 0.70±0.2 g. In the 3-tag trial, 30% of male zebrafish retained all tags, 30% lost their pelvic tag and one of the dorsal tags and 40% lost their pelvic tag but retained their dorsal tags. Growth was comparable to control fish; the average pre tagging weight was 0.45±0.02 g, male zebrafish with tags weighed 0.48±0.02 g and control fish weighed 0.47±0.02 g. Sixty percent of female zebrafish retained all tags, and 40% lost the pelvic tag but retained their dorsal tags. Growth was comparable to control fish where the average pre-tagging weight was 0.69±0.05 g, female zebrafish with tags weighed 0.75±0.5 g and control fish weighed 0.70±0.2 g. Tagging time for two tags/fish was 38±8 seconds and for three tags it increased to 46±4 seconds. In summary, multiple tagging did not affect the growth and survival of adult zebrafish. Dorsal tags were most reliably retained.

### Trial 6: Survival of rag1^−/−^ zebrafish

VIE tags can be safely used to tag immunodeficient *rag1^−/−^* zebrafish; all tagging treatments had 100% survival.

## Discussion

In laboratories that maintain zebrafish lines that are phenotypically indistinguishable, the use of VIE tags can be useful. In a laboratory setting, tagging is essential when one needs to identify fish of different genotypes, to run longitudinal monitoring of individual traits like weight gain and growth, or to repeat assays with the same individual at discrete points in time. The challenging characteristic of zebrafish for tagging purposes is their small size. Tagging methods we have used with other fish species include anchor tags that are punctured into the epidermis of the body or a fin (percutaneous), visible implant (VI) alpha numeric tags that are implanted under the skin (subcutaneous) and passive inducer transmitters (PIT) tags that are invasively placed under the skin or within the abdominal cavity (internal). Anchor and pit tags are too large for use in zebrafish. The injection force of skin tattooing, a less invasive marking method, killed channel catfish fry the size of zebrafish (unpublished data). Recently, RFID microtags have been evaluated in zebrafish (Cousin et al., 2012). These tags although much smaller than regular PIT tags are rather invasive and costly when compared to the VIE tagging method. The RFID microtags offer a large number of code combinations, an advantage when large numbers of individual tags are needed. As demonstrated in trial 5 up to 3 VIE tags can be injected into adult zebrafish, increasing the possibilities for individual tagging.

The VIE method has been successfully used with small fish such as mummichogs (Skinner et al., 2006), small perch (*Perca fluviatilis*) and common bully (*Gobiomorphus cotidianus*) (Goldsmith et al., 2003). Sites that were more easily injected in larger fish were difficult to inject in zebrafish. However, following injection, our zebrafish, especially adults, had high survival and good tag retention times. Reduced tag retention was observed in juvenile zebrafish and fish injected with multiple tags. Application of VIE tags in species that will remain small offer fewer challenges than tagging fish that will grow substantially during the use of the tag (Northwest Marine Technology, Inc., 2006, ‘Tagging small fish with visible implant elastomer’). In small tropical fish (<20 mm) the investigator's experience with the technique was directly correlated with elastomer retention and visibility. When administered by an experienced investigator the VIE tagging method did not affect survival and growth of small fish (Frederick, 1997), as we also observed.

Long-term elastomer tag detection rates in fingerling rainbow trout were low (29 to 33%) approximately 2 years after the tagging (Close and Jones, 2002). Our long term study showed that VIE tags were still visible after one year. This is the approximate time zebrafish are held at most research facilities. The VIE tag project manual states that quantities as small as 0.1 mL of elastomer could be prepared (Northwest Marine Technology, Inc., 2008, ‘Visible implant elastomer tag project manual’). We determined that up to 50 zebrafish can be tagged with 0.1 mL of elastomer within 30 minutes. Once elastomer is prepared it needs to be used within 1 hour before polymerization occurs (Northwest Marine Technology, Inc., 2008, ‘Visible implant elastomer tag project manual’). Mixing the smallest quantity shortly before injection of fish minimized wastage of elastomer.

In the current zebrafish study, the dorsal and pectoral fin bases offered a good compromise between ease of injection, visibility and tag retention. Differentiations of color visibility and tag retention rates by anatomical location on fish have been specifically addressed in several studies (Dewey and Zigler, 1996; Farooqi and Morgan, 1996; Goldsmith et al., 2003; Gallagher and Hutchinson, 2004). In Silver perch, *Bidyanus bidyanus*, and Australian bass, *Macquaria novemaculeata*, tag visibility was good in most locations (Gallagher and Hutchinson, 2004). In both species the least visible tags were front dorsal tags for which less than 90% were visible after 8 months (Gallagher and Hutchinson, 2004). The best tag locations for both species were behind the dorsal fin and adjacent to the anal fin (Gallagher and Hutchinson, 2004). Combinations of four colors and three locations were used with perch and common bully (Goldsmith et al., 2003). In perch, tag retention was 100% throughout the study, but in bully, originally high tag retention decreased to 72% after 125 days (Goldsmith et al., 2003). In barbell, *Barbus barbus*, VIE tags in the postorbital adipose tissue were retained at a lower rate (48%) than in the scalp and caudal and anal fin bases (82%) (Farooqi and Morgan, 1996). In bluegill, however, none of the scalp tags were retained (Dewey and Zigler, 1996).

The most visible color to use depends on the natural pigmentation of the fish being tagged (Close and Jones, 2002; Walsh and Winkelman, 2004; Astorga et al., 2005; Curtis, 2006). Red was the best color to use in seabream (Astorga et al., 2005), and red and orange were the best in seahorses *Hippocampus guttulatus* (Curtis, 2006). Pink and red were the most visible in zebrafish, and orange and yellow also worked well. However, in zebrafish, yellow and orange were difficult to distinguish from each other, as were pink and red. In seahorses it was difficult to distinguish orange tags from red or pink tags and green tags from yellow tags (Curtis, 2006).

Many mutant zebrafish strains are used in experimental settings so it was important to evaluate VIE tagging in mutant fish. Our T and B cell deficient mutant zebrafish where handled the same way as wild-type zebrafish and VIE injection was safe to use on immune-compromised *rag1^−/−^* fish. We recommend determining survival in mutant zebrafish strains before the use of VIE tags in experiments.

Overall, our results suggest that the VIE tagging method is suitable for zebrafish, as young as 1 month old. Pink VIE tags injected at the dorsal fin base were retained and remained the most visible long term. Visible implant elastomer tagging provides excellent identification resources for zebrafish and other small fish in in-door research facilities.
